# Left atrial late gadolinium enhancement following external beam radiation for lymphoma: a potential model for exploring radiation-related heart disease

**DOI:** 10.1186/1532-429X-14-S1-P187

**Published:** 2012-02-01

**Authors:** Alexis Harrison, Kavitha Damal, Nathan S Burgon, Mark M Haslam, Martha Glenn, Christopher McGann, Nassir F Marrouche, Brent Wilson

**Affiliations:** 1 Cardiology, University of Utah, Salt Lake City, UT, USA; 2 Comprehensive Arrhythmia Research and Management (CARMA) Center, Salt Lake City, UT, USA; 3 Huntsman Cancer Institute at University of Utah, Salt Lake City, UT, USA

## Summary

We are able to detect subclinical post-irradiation changes to the heart with left atrial late gadolinium enhanced magnetic resonance imaging (LGE-MRI).

## Background

There are over 11 million cancer survivors, yet many long-term cancer survivors experience lasting changes including cardiac functional and anatomical abnormalities following external beam radiation and chemotherapy. One of the well-established pathological and histological sequelae of radiation exposure to the heart is intense post-irradiation injury with fibrotic changes. Here, we have explored the ability to apply LGE to identify the extent of left atrial uptake after radiation therapy.

## Methods

A total of 20 patients were enrolled in this study; 10 patients (ages 29 to 64 years) who had survived 9.2 ± 25 years after thoracic external beam radiation for lymphoma and 10 control patients (ages 55 to 66 years) recruited from the University colonoscopy center. All patients underwent a MRI study including cine imaging, left ventricular LGE, and high-resolution LGE imaging of the left atrium. The extent of late gadolinium enhancement was calculated as a relative percent of total left atrial wall using a threshold-based algorithm based on pixel intensity distribution.

## Results

**Figure 1:**
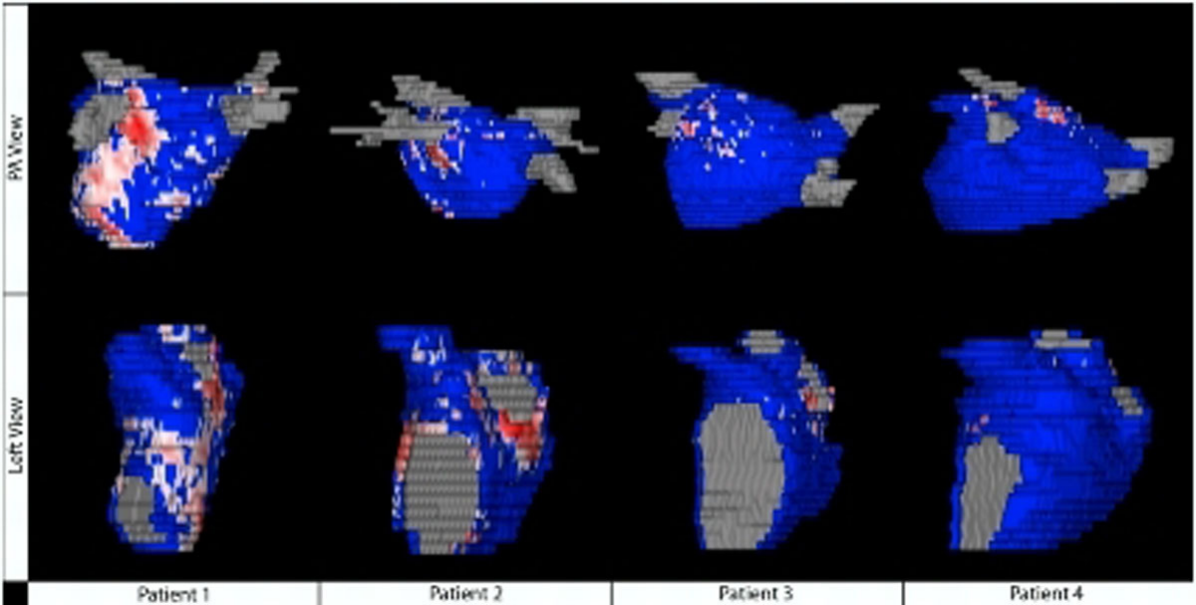
Comparison of late gadolinium enhancement in the left atrium in patients with and without external beam radiotherapy. Posteroanterior (above) and left lateral (below) views of the atrium of LGE -MRI in patients with a history of radiation (patients 1 and 2) and in control patients (patients 3 and 4) demonstrating increased signal with the radiation exposure. Patients 1 and 2 showed enhancement in 17.15% and 7.72% of the left atrium, respectively, while Patients 3 and 4 showed enhancement in 1.80% and 0.87%, respectively, of the left atrium.

**Figure 2:**
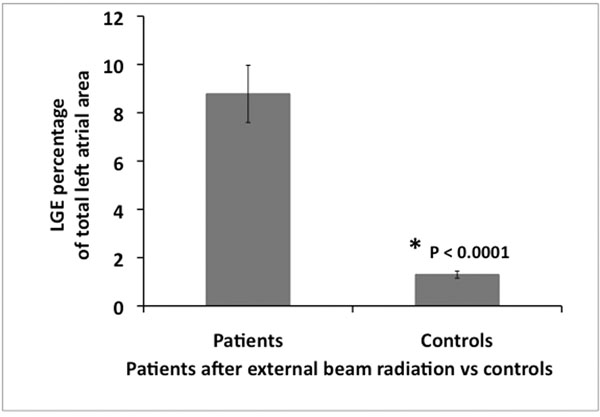
Comparison of LGE percentage of the total left atrial area in patients with and without external beam radiation.

As seen in Figures 1 and 2, the extent of LGE as a relative percentage of the total left atrial area was significantly greater in patients who had a history of external beam radiation than controls (8.78 ± 3.75% vs. 1.30 ± 0.47%, p = 0.0001). Excluding the two patients who had external beam radiation over 10 years ago (prior to modern heart shielding improvements), there was still a significantly greater percentage of left atrial enhancement in patients with external beam radiation than in controls (7.08 ± 0.92% vs. 1.30 ± 0.47%, P = 0.0001).

## Conclusions

Patients with thoracic radiation for lymphoma have greater LGE reflecting radiation-induced injury than in an older group of controls without thoracic radiation therapy. LGE-MRI shows promise for finding and screening for prevalent myocardial tissue changes in this group of patients. Further studies with larger patient populations post thoracic radiation therapy with and without chemotherapy (as chemotoxic effects also include fibrotic changes) and with longitudinal follow up would be useful for correlation with external beam radiation dosimetry and the development of future cardiovascular events in long-term cancer survivors.

## Funding

University of Utah Seed Grant.

